# 4-(Ammonio­meth­yl)pyridinium dichloride

**DOI:** 10.1107/S1600536808034405

**Published:** 2008-10-25

**Authors:** Meher El Glaoui, Riadh Kefi, Olfa Amri, Erwann Jeanneau, Cherif Ben Nasr

**Affiliations:** a Laboratoire de Chimie des matériaux, Faculté des sciences de Bizerte, 7021 Zarzouna, Tunisia; b Universtié Lyon1, Centre de Diffractométrie Henri Longchambon, 43 boulevard du 11 Novembre 1918, 69622 Villeurbanne Cedex, France

## Abstract

The title compound, C_6_H_10_N_2_^2+^·2Cl^−^, contains a network of 4-(ammonio­meth­yl)pyridinium cations and chloride anions which are inter­connected by N—H⋯Cl hydrogen bonds. The crystal packing is also influenced by inter­molecular π–π stacking inter­actions between identical anti­parallel organic cations with a face-to-face distance of *ca* 3.52 Å.

## Related literature

For common applications of this type of complex, see: Schmidtchen & Berger, (1997 ▶); Pajewski *et al.* (2004 ▶); Sessler *et al.* (2003 ▶); Ilioudis *et al.* (2000 ▶). For structure cohesion, see: Bernstein *et al.*, (1995 ▶); Jin *et al.*, 2005 ▶. For discussion of the C—N—C angle, see: Krygowski *et al.* (2005 ▶). For bond-length data, see: Oueslati *et al.* (2006 ▶).
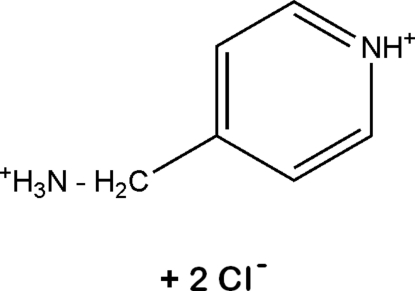


## Experimental

### 

#### Crystal data

C_6_H_10_N_2_^2+^·2Cl^−^
*M* *_r_* = 181.06
Triclinic, 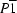

*a* = 7.257 (2) Å
*b* = 7.339 (3) Å
*c* = 8.752 (1) Å
α = 79.14 (3)°
β = 70.94 (4)°
γ = 70.19 (3)°
*V* = 412.9 (2) Å^3^
*Z* = 2
Mo *K*α radiation
μ = 0.71 mm^−1^
*T* = 293 K
0.16 × 0.15 × 0.12 mm

#### Data collection

Enraf–Nonius CAD-4 diffractometer
Absorption correction: none
3311 measured reflections
1995 independent reflections
1670 reflections with *I* > 2σ(*I*)
*R*_int_ = 0.014
2 standard reflections every 400 reflections intensity decay: 4%

#### Refinement

*R*[*F* ^2^ > 2σ(*F* ^2^)] = 0.029
*wR*(*F* ^2^) = 0.030
*S* = 1.06
1609 reflections
91 parameters
H-atom parameters constrained
Δρ_max_ = 0.29 e Å^−3^
Δρ_min_ = −0.20 e Å^−3^

### 

Data collection: *CAD-4 EXPRESS* (Straver, 1992 ▶); cell refinement: *CAD-4 EXPRESS*; data reduction: *RC93* (Watkin *et al.*, 1994 ▶); program(s) used to solve structure: *SIR97* (Altomare *et al.*, 1999 ▶); program(s) used to refine structure: *CRYSTALS* (Betteridge *et al.*, 2003 ▶); molecular graphics: *CAMERON* (Watkin *et al.*, 1996 ▶); software used to prepare material for publication: *CRYSTALS*.

## Supplementary Material

Crystal structure: contains datablocks global, I. DOI: 10.1107/S1600536808034405/bg2217sup1.cif

Structure factors: contains datablocks I. DOI: 10.1107/S1600536808034405/bg2217Isup2.hkl

Additional supplementary materials:  crystallographic information; 3D view; checkCIF report

## Figures and Tables

**Table 1:** Hydrogen-bond geometry (Å, °)

*D*—H⋯*A*	*D*—H	H⋯*A*	*D*⋯*A*	*D*—H⋯*A*
N1—H1⋯Cl1^i^	0.83	2.36	3.084 (2)	146
N2—H8⋯Cl1	0.89	2.28	3.160 (3)	171
N2—H9⋯Cl2^ii^	0.90	2.23	3.126 (2)	173
N2—H10⋯Cl2^iii^	0.89	2.37	3.190 (2)	152

Symmetry codes: (i) 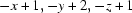; (ii) 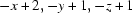; (iii) 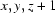.

**Fig. 1.**
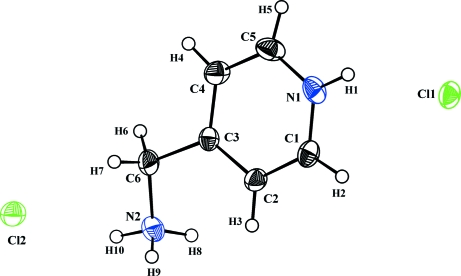
A view of (I), showing 40% probability displacement ellipsoids and arbitrary spheres for the H atoms.

**Fig. 2.**
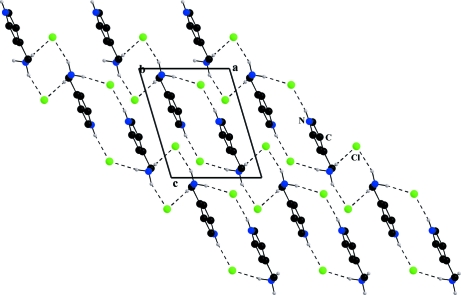
Crystal structure of C6H10Cl2N2 viewed along b axis showing that the molecules crystallize in a ring motif.

**Fig. 3.**
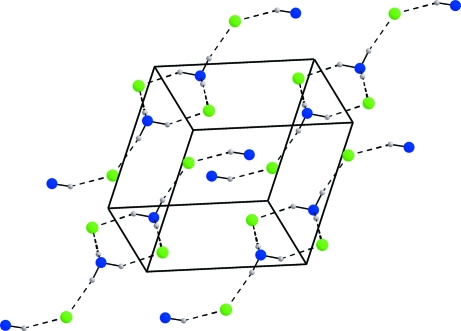
Perspective view of (I) showing four chains across the unit cell at b = 1/2 in the [111] direction

## supplementary crystallographic information

### Comment

The coordination chemistry of anions was the starting point for the development of new compounds having many practical and potential applications in various fields, such as supramolecular chemistry (Schmidtchen and Berger, 1997) and biochemical processes (Pajewski *et al.*, 2004). Moreover, halide anions have been successfully used to assemble double-helical motifs of various molecules containing aromatic groups, with π-stacking interactions within the helices (Sessler *et al.*, 2003). These anions can be useful for such applications because of the high flexibility of their coordination (Ilioudis *et al.*, 2000). Here, a new member of this family, the title compound (C_6_H_10_Cl_2_N_2_), is presented, which has been  obtained during our studies of the preparation of new organic hydrochloride compounds. As shown in Fig. 1, to ensure charge balance  the organic species is doubly protonated at N1 and N2. Thus, the structure consists  essentially of an 4-(ammoniomethyl)pyridinium cations and two Cl^-^ anions, associated in a hydrogen-bonded network. The Cl^-^ anions and the antiparallel pair of organic cations associate each other *via* hydrogen-bonding interactions to construct a convoluted hydrogen-bonded chain network which runs along the [111] direction at b = ^1^/_2_ (Fig. 3). This chain is made up by a four-membered donor-acceptor ring, involving two Cl atoms, fused along the N—H···Cl hydrogen bond (Fig. 2). These intermolecular hydrogen bonds generate edge-fussed [*R*_2_^4^(8) and *R*_2_^4^(20)] motifs (Bernstein *et al.*, 1995). When viewed in perspective, the molecules chains have a marked zigzag structure and somewhat resembles a helix. As can be seen in Fig.2, the neighbouring pyridinyl rings run parallel in  opposite directions and stack each other by turns in a face-to-face mode. The nearest centroid-centroid distance is 3.52 Å, less than 3.8 Å, a usually  acceptad maximum value for π-π interactions (Jin *et al.*, 2005). An examination of the organic moiety geometrical features shows that the atoms building the pyridinyl ring have a good coplanarity and they form a conjugated plane with average deviation of 0.005 Å). The mean value of C—C and N—C bond lengths are 1.381 (2) and 1.332 (2) Å) which are between that of a single bond and a double bond and agree with those in the literature (Oueslati *et al.*, 2006). However, it is worth noticing that the C—N—C angles of pyridine are very sensitive to protonation (Krygowski *et al.*, 2005). A pyridinium cation always possesses an expanded angle of C—N—C in comparison with the parent pyridine. The  C1—N1—C5 angle [122.3 (2) °] is consistent with the type of pyridinium cation. In fact, the protonation of the nitrogen atom N1 decreases its electronegativity; hence the corresponding C—N—C angles becomes larger.

### Experimental

An aqueous 1*M* HCl solution and 4-(amminomethyl)pyridine in a 2:1 molar ratio were mixed and dissolved in sufficient ethanol. Crystals of (I) grew as the ethanol evaporated at 293 K over the course of a few days.

### Refinement

The refinement was carried out with Iσ(I)>3 and a sinθ/λ>0.01 to get rid of the reflections in the vicinity of the beamstop. The refinement was thus carried out using 1609 reflections (out of the 1995 independent ones). The R value reported corresponds to the recomputed value with a 2σ cutoff (SHELX like).

The H atoms were all located in a difference map, but those attached to carbon atoms were repositioned geometrically. The H atoms were initially refined with soft restraints on the bond lengths and angles to regularize their geometry (C—H in the range 0.93–0.98, N—H in the range 0.86–0.89 and O—H = 0.82 Å) and *U*_iso_(H) (in the range 1.2–1.5 times *U*_eq_ of the parent atom), after which the positions were refined with riding constraints.

### Crystal data

**Table:**

C_6_H_10_N_2_^2+^·2Cl^−^	*Z* = 2
*M**_r_* = 181.06	*F*(000) = 188
Triclinic, *P*1	*D*_x_ = 1.456 Mg m^−^^3^
Hall symbol: -P 1	Mo *K*α radiation, λ = 0.71073 Å
*a* = 7.257 (2) Å	Cell parameters from 25 reflections
*b* = 7.339 (3) Å	θ = 9–11°
*c* = 8.752 (1) Å	µ = 0.71 mm^−^^1^
α = 79.14 (3)°	*T* = 293 K
β = 70.94 (4)°	Block, colorless
γ = 70.19 (3)°	0.16 × 0.15 × 0.12 mm
*V* = 412.9 (2) Å^3^	

### Data collection

**Table:**

Enraf–Nonius CAD-4 diffractometer	θ_max_ = 28.0°, θ_min_ = 2.5°
graphite	*h* = −9→9
ω/2θ scans	*k* = −9→9
3311 measured reflections	*l* = −5→11
1995 independent reflections	2 standard reflections every 400 reflections
1670 reflections with *I* > 2σ(*I*)	intensity decay: 4%
*R*_int_ = 0.014	

### Refinement

**Table:**

Refinement on *F*	Primary atom site location: structure-invariant direct methods
Least-squares matrix: full	Hydrogen site location: inferred from neighbouring sites
*R*[*F*^2^ > 2σ(*F*^2^)] = 0.030	H-atom parameters constrained
*wR*(*F*^2^) = 0.030	[*w*eight] = 1.0/[A_0_*T_0_(x) + A_1_*T_1_(x) ··· + A_n-1_]*T_n-1_(x)] where A_i_ are the Chebychev coefficients listed belo*w* and x = *F* /*F*max W = [*w*eight] * [1-(delta*F*/6*sigma*F*)^2^]^2^ A_i_ are: 0.823 0.257 0.531
*S* = 1.06	(Δ/σ)_max_ = 0.001
1609 reflections	Δρ_max_ = 0.29 e Å^−^^3^
91 parameters	Δρ_min_ = −0.20 e Å^−^^3^
0 restraints	

### Fractional atomic coordinates and isotropic or equivalent isotropic displacement parameters (Å^2^)

**Table:**

	*x*	*y*	*z*	*U*_iso_*/*U*_eq_	
Cl1	0.36975 (6)	0.70916 (5)	0.85091 (4)	0.0357	
Cl2	0.85118 (6)	0.60355 (5)	0.28807 (4)	0.0385	
H1	0.6965	1.2683	0.3992	0.0408*	
H2	0.7426	0.9613	0.4009	0.0420*	
H3	0.7984	0.7600	0.6294	0.0385*	
H4	0.7325	1.2354	0.8318	0.0427*	
H5	0.6824	1.4162	0.5939	0.0458*	
H6	0.7155	0.9365	1.0028	0.0427*	
H7	0.9427	0.8393	0.9126	0.0426*	
H8	0.6516	0.6757	0.9274	0.0509*	
H9	0.8719	0.5860	0.8743	0.0510*	
H10	0.7711	0.6183	1.0429	0.0511*	
N1	0.71013 (19)	1.20012 (18)	0.48454 (13)	0.0347	
N2	0.76974 (19)	0.66782 (17)	0.94222 (14)	0.0340	
C1	0.7421 (2)	1.0097 (2)	0.49055 (16)	0.0330	
C2	0.7738 (2)	0.8922 (2)	0.62759 (16)	0.0309	
C3	0.76881 (19)	0.97525 (19)	0.76000 (15)	0.0263	
C4	0.7352 (2)	1.1743 (2)	0.74770 (16)	0.0350	
C5	0.7057 (3)	1.2852 (2)	0.60805 (18)	0.0401	
C6	0.8048 (2)	0.8595 (2)	0.91467 (16)	0.0334	

### Atomic displacement parameters (Å^2^)

**Table:**

	*U*^11^	*U*^22^	*U*^33^	*U*^12^	*U*^13^	*U*^23^
Cl1	0.0459 (2)	0.04261 (19)	0.02508 (16)	−0.01833 (15)	−0.01593 (13)	0.00223 (12)
Cl2	0.0537 (2)	0.03529 (18)	0.02893 (17)	−0.01461 (15)	−0.01409 (14)	−0.00186 (13)
N1	0.0390 (6)	0.0379 (6)	0.0203 (5)	−0.0065 (5)	−0.0087 (4)	0.0048 (4)
N2	0.0397 (6)	0.0337 (6)	0.0276 (5)	−0.0107 (5)	−0.0136 (5)	0.0062 (4)
C1	0.0371 (7)	0.0430 (8)	0.0218 (6)	−0.0148 (6)	−0.0090 (5)	−0.0036 (5)
C2	0.0393 (7)	0.0304 (6)	0.0266 (6)	−0.0136 (5)	−0.0116 (5)	−0.0011 (5)
C3	0.0265 (6)	0.0314 (6)	0.0214 (5)	−0.0101 (5)	−0.0071 (4)	0.0003 (5)
C4	0.0487 (8)	0.0343 (7)	0.0220 (6)	−0.0146 (6)	−0.0070 (6)	−0.0037 (5)
C5	0.0564 (9)	0.0271 (7)	0.0287 (7)	−0.0076 (6)	−0.0074 (6)	−0.0010 (5)
C6	0.0420 (7)	0.0359 (7)	0.0262 (6)	−0.0129 (6)	−0.0162 (5)	0.0024 (5)

### Geometric parameters (Å, °)

**Table:**

H3—C2	0.923	H9—N2	0.899
H2—C1	0.919	N1—C1	1.331 (2)
H5—C5	0.909	N1—C5	1.333 (2)
H8—N2	0.890	N2—C6	1.4750 (19)
H7—C6	0.955	C6—C3	1.5065 (18)
H6—C6	0.961	C1—C2	1.3750 (19)
H1—N1	0.831	C3—C2	1.3929 (18)
H4—C4	0.923	C3—C4	1.386 (2)
H10—N2	0.890	C5—C4	1.371 (2)
			
H1—N1—C1	118.7	H2—C1—N1	117.8
H1—N1—C5	118.6	H2—C1—C2	122.2
C1—N1—C5	122.62 (12)	N1—C1—C2	120.03 (13)
H9—N2—H8	109.1	C6—C3—C2	123.50 (12)
H9—N2—H10	107.4	C6—C3—C4	117.95 (12)
H8—N2—H10	109.7	C2—C3—C4	118.52 (12)
H9—N2—C6	109.8	H5—C5—N1	116.9
H8—N2—C6	111.9	H5—C5—C4	123.6
H10—N2—C6	108.9	N1—C5—C4	119.51 (14)
N2—C6—H6	109.7	C3—C2—C1	119.26 (13)
N2—C6—H7	108.0	C3—C2—H3	121.6
H6—C6—H7	108.4	C1—C2—H3	119.2
N2—C6—C3	114.31 (11)	C3—C4—H4	121.5
H6—C6—C3	107.3	C3—C4—C5	120.05 (13)
H7—C6—C3	108.9	H4—C4—C5	118.4

### Hydrogen-bond geometry (Å, °)

**Table:**

*D*—H···*A*	*D*—H	H···*A*	*D*···*A*	*D*—H···*A*
N1—H1···Cl1^i^	0.83	2.36	3.084 (2)	146
N2—H8···Cl1	0.89	2.28	3.160 (3)	171
N2—H9···Cl2^ii^	0.90	2.23	3.126 (2)	173
N2—H10···Cl2^iii^	0.89	2.37	3.190 (2)	152

Symmetry codes: (i) −*x*+1, −*y*+2, −*z*+1; (ii) −*x*+2, −*y*+1, −*z*+1; (iii) *x*, *y*, *z*+1.
